# Rhabdomyolysis in a Patient Taking Both Oxandrolone for Bodybuilding and Methamphetamine

**DOI:** 10.7759/cureus.1843

**Published:** 2017-11-13

**Authors:** Michael Krzyzak, Natarajan Elangovan

**Affiliations:** 1 Medicine, Staten Island University Hospital; 2 Department of Psychiatry, Staten Island University Hospital

**Keywords:** rhabdomyolisis, methamphetamine, polysubstance

## Abstract

Nonprescription drug use is increasingly prevalent in the United States. We report a case of a 31-year-old male who presented with hallucinations and was found to have rhabdomyolysis. He was consuming oxandrolone for six weeks and ingested methamphetamine the night prior to presentation. With supportive treatment, including intravenous hydration, the patient’s mental status returned to baseline and rhabdomyolysis resolved. Our case illustrates the need to understand the interaction between different illicit substances. More research needs to be done to further understand the reactions between different medications as patients consume different combinations of substances.

## Introduction

Polysubstance use is becoming a common issue when uncovering unknown side effects of nonprescription medication. In the United States, 6.3% of people have taken nonprescription drugs. In the 18- to 29-year-old group, the prevalence of polysubstance use is 13.5% [1]. Herein, we report a patient that presented with acute delusion found to be in rhabdomyolysis after consuming methamphetamine while using testosterone for bodybuilding.

## Case presentation

A 31-year-old male presented to the emergency room with complaints of unusual behavior at work. He noted that while working at a metal shop, he heard voices and saw unusual figures. The night prior, he was watching a sporting event with friends and took 3,4-methylenedioxymethamphetamine (MDMA). He also uses oxandrolone injections for muscle building. Oxandrolone was started approximately six weeks prior to presentation. He takes no other medication or supplementation.

On presentation, vitals were normal. Mental status was positive for auditory and visual hallucinations. He was confused as per his family but was oriented fully. Physical examination was otherwise unremarkable. 

Laboratory findings were significant for a creatine kinase level of 1893 IU/L, creatine kinase-MB of 43.0 ng/mL, aspartate transaminase (AST) 137 IU/L, and alanine transaminase (ALT) 212 IU/L. He received intravenous hydration and haloperidol for agitation. He was admitted to the telemetry unit. Within 24 hours of hospitalization, the mental status returned to baseline. Creatine kinase trended down to 841 IU/L and creatine kinase-MB to 16.5 IU/L. He was advised not to continue hormone injection or drug use. He was discharged home to follow up with his primary care physician.

## Discussion

Between one and three million people in the United States are on hormonal supplements for bodybuilding [2]. Studies illustrate that up to 5% of high school students have tried performance-enhancing substances [2].

Medical indications for anabolic steroid use include hypogonadism, catabolic disorders as muscle wasting, growth retardation, tissue healing, cachexia, osteoporosis, aplastic anemia, virile climacteric period, and hepatic carcinoma. Athletes consume steroids for its anabolic properties, which include promoting protein synthesis, positive nitrogen balance, muscle growth, increasing calcium uptake, stimulation of skeletal growth, erythropoiesis, the percentual decrease of body fat, and V-shaped bodybuilding [3].

Anabolic steroid use in bodybuilders is known to cause many side effects. Effects include venous thromboembolism, arterial thromboembolism, disc herniation, hypertrophic cardiomyopathy, dilated cardiomyopathy, hepatocellular carcinoma, renal failure, depression, and suicide thoughts [4]. Oxandrolone is metabolized by 17α-alkylation, which is hepatotoxic. The elevation in AST and ALT noted in our case can be explained by end-organ damage secondary to oxandrolone [4].

Methamphetamine is known as a psychomotor stimulant, and psychosis is known to last up to several weeks. Effects include violent behavior and paranoid psychosis. Its mechanism of action is directing its effect on the manufacture of dopamine and serotonin causing an alteration in sleep, sexual function, movement disorders, and schizophrenia [5].

Methamphetamine is metabolized by the liver via demethylation, primarily demethylated by cytochrome P 450 2D (CYP2D6) [6-7]. Similarly, oxandrolone is also metabolized by the liver; unlike other testosterone derivatives, it takes longer to be deactivated by hydroxylation and sulfation [8]. The combination of the two medications produces a synergistic effect causing rhabdomyolysis. It is supported by their common metabolic pathway and both inactivated products are later excreted in urine.

Studies have evaluated the interaction between anesthetic agents and illicit substances, but there is no research on the effects of interaction between illicit substances [9].

## Conclusions

Studies have evaluated the interaction between numerous medications, but more research needs to be performed in order to understand the interaction between illicit substances. Given the polysubstance use in the discussed case, clinicians should be aware of polysubstance use in the community and its effects. As demonstrated in this case, combinations of different illicit substances can result in exaggerated effects of either substance alone and produce dangerous results.
